# Transvaginal Appendectomy in Morbidly Obese Patient

**DOI:** 10.1155/2014/368640

**Published:** 2014-11-20

**Authors:** Mehmet Ali Yagci, Cuneyt Kayaalp, Mustafa Ates

**Affiliations:** Department of Surgery, Turgut Ozal Medical Center, Inonu University, 44315 Malatya, Turkey

## Abstract

*Introduction.* Laparoscopic appendectomy has significant benefits in obese patients. However, morbid obesity can be accepted as an exclusion criterion for natural orifice transluminal endoscopic surgery (NOTES). Here, we present a transvaginal appendectomy in a 66-year-old morbidly obese (BMI 36 kg/m^2^, ASA III) patient. *Case and Technique.* Acute appendicitis was suspected based on history, physical examination, laboratory tests, and ultrasound findings. During laparoscopic surgery, a 5 mm trocar was inserted through the umbilicus and a 5 mm telescope was placed. A 12 mm trocar and a 5 mm grasper were inserted separately through the posterior fornix of the vagina under laparoscopic guidance. The appendix was divided with an endoscopic stapler through the transvaginal 12 mm trocar and removed from the same trocar. The operating time was 75 minutes with minimal blood loss (<10 mL). The patient was discharged 16 hours after surgery uneventfully and she did not require any analgesic administration. *Conclusion.* To the best of our knowledge, this is the first clinical case that focuses on the transvaginal appendectomy at morbid obesity. We can say that morbid obesity does not constitute an obstacle for treatment of acute appendicitis by transvaginal endoscopic surgery.

## 1. Introduction

Acute inflammation of the appendix is probably as old as humankind, and appendectomies were first described almost 130 years ago [1]. Appendectomy methods have improved in parallel to technological developments, and laparoscopic appendectomy came into clinical practice 30 years ago [2]. When laparoscopic appendectomies were first utilized, obesity was a relative contraindication, although in time it was demonstrated that laparoscopic appendectomy was more valuable for obese patients than for patients of normal weight [3]. Recently, natural orifice endoluminal surgery (NOTES) was introduced as a new approach that allows surgical procedures mainly through natural orifices, such as the mouth, anus, or vagina [4, 5]. It aims to avoid or decrease the use of incisions on the body's surface. This potential advantage could help to reduce surgical pain, decrease analgesic requirements, shorten recovery times, avoid hernia formation and adhesions, and eliminate surgical site infections and visible scarring [4]. As of today, more than 100 transvaginal appendectomy cases have been reported. All the case series accepted morbid obesity as an exclusion criterion for transvaginal appendectomy. The aim of this case report was to describe the initial clinical experience of transvaginal appendectomy in a morbidly obese patient and to investigate its feasibility and surgical outcome.

## 2. Case and Technique

**Figure 1:**
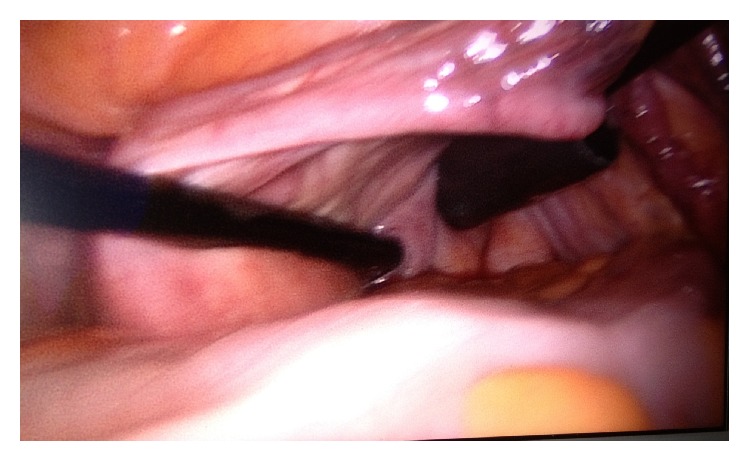
Transvaginal 12 mm trocar and a separate 5 mm grasper (without trocar) placement under laparoscopic guidance. View of posterior fornix.

**Figure 2:**
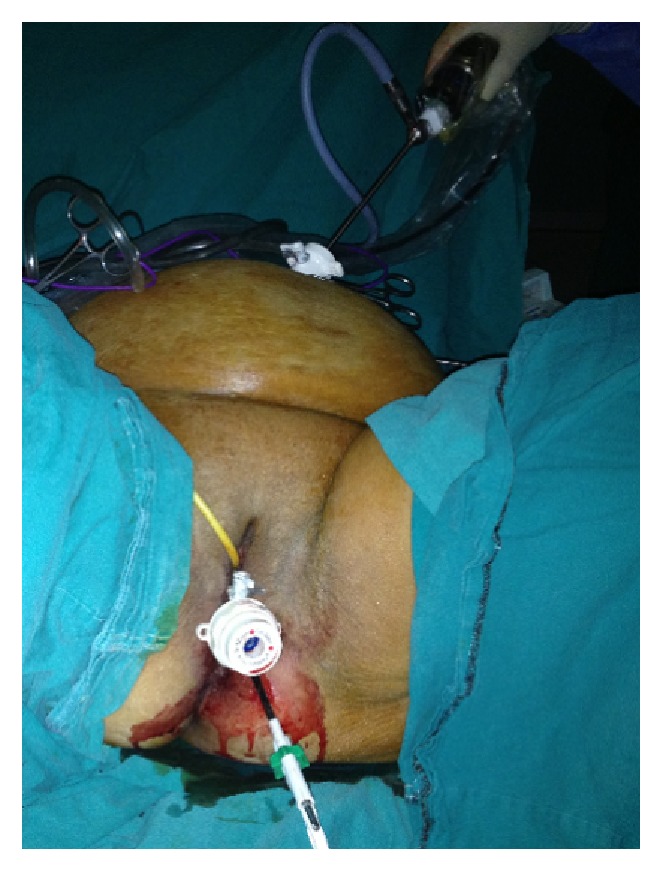
Transumbilical 5 mm, 30° laparoscope and perineal view.

**Figure 3:**
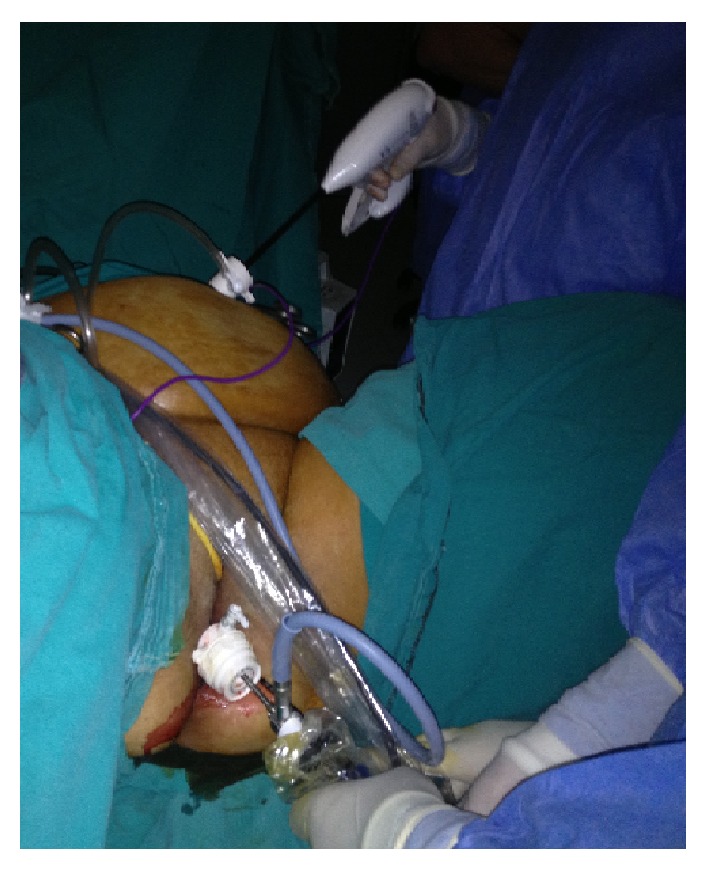
Division of mesoappendix through the umbilical port by Ligasure (5 mm).

A 66-year-old female patient was admitted to the emergency department for abdominal pain at the right lower quadrant persisting for two days. Her medical history showed significant chronic obstructive pulmonary disease and coronary artery disease. Her body mass index (BMI) was 36 kg/m^2^, and her American Society of Anesthesiologists (ASA) score was III. Based on history taking and physical examination, acute appendicitis was suspected; thus, laboratory tests and abdominal ultrasonography were performed. The blood test revealed that leucocytes were elevated to 15600/mm^3^, and abdominal ultrasound demonstrated that the appendix was thickened to 8 mm, thus confirming acute appendicitis. We decided on a minimally invasive approach using transvaginal appendectomy. The patient fully consented to the operation. Half an hour prior to surgery, a second-generation antibiotic (cefazolin 1 gr) was administrated intravenously. Under general anesthesia, the patient was placed in the lithotomy position. The abdomen, pelvis, and vaginal canal were disinfected with povidone iodine. A 5 mm skin incision was made into the umbilicus, and after a pneumoperitoneum was created with a Veress needle, a 5 mm trocar was inserted. A 5 mm laparoscopic camera (30°) was used for abdominal exploration and the diagnosis was confirmed. A 12 mm trocar and a 5 mm grasper without a trocar were inserted separately through the posterior fornix of the vagina under laparoscopic guidance (Figure 1). The reason for the transvaginal grasper insertion without trocar was the proximity of the surgical instruments at the perineum (Figure 2). At first we tried to keep the laparoscope at the umbilical trocar. However hanging and division maneuvers from the same access (unidirectional) were not comfortable and did not provide a good laparoscopic view. So, we moved the laparoscope to the vaginal trocar. This obtained more than 90° angle between the hanging and dividing instruments with a better view. After moving the camera to the vaginal trocar, the distal end of the appendix was held with the vaginal grasper and the mesoappendix was divided by Ligasure (ForceTriad, Covidien, Boulder, CO, USA) through the umbilical trocar (Figure 3). The laparoscope was again moved to umbilical trocar and an endoscopic stapler (EndoGIA, Covidien, Mansfield, MA, USA) from the transvaginal trocar was used for division of the appendix from the cecum. The appendix was placed in a specimen bag and removed from the same vaginal port. After inspection for hemostasis and bowel integrity, the colpotomies were closed with interrupted sutures using 1.0 Vicryl. No drain was used, and a vaginal pack dressing was applied.

**Figure 4:**
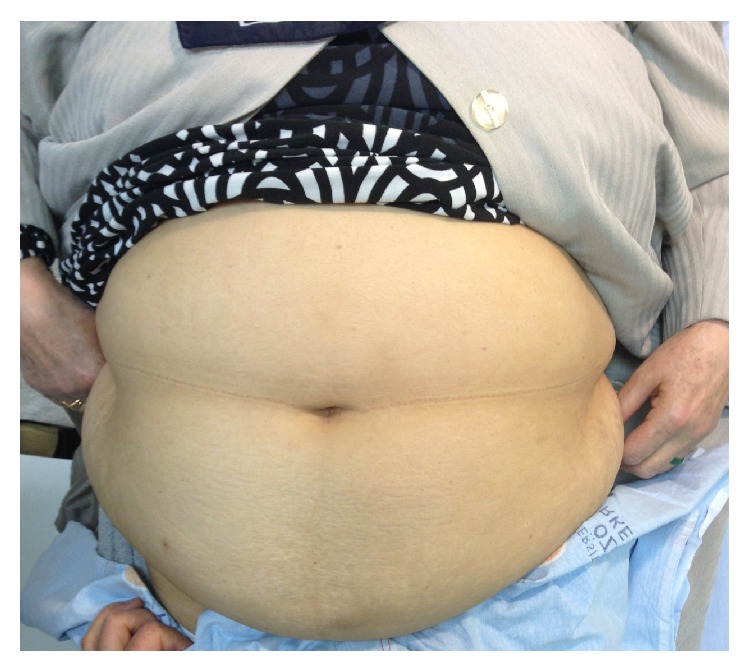
Postoperative appearance of the abdomen.

The operating time was 75 minutes with minimal blood loss (<10 mL). The patient began drinking water at night, and she was discharged 16 hours after surgery without wound infection, fever, pain, urinary difficulty, or any other complication. Analgesics were not administrated during the postoperative period. Vaginal pack was removed on day one and no more packing was required. At the one-month ambulatory follow-up, the abdominal and vaginal wounds had healed well, and the patient had no complaints (Figure 4). The histopathological examination confirmed the diagnosis of acute appendicitis.

## 3. Discussion

Although laparoscopic surgery has several advantages over open surgery, it is not pain-free, and there are still wound-related early and late complications, such as hernias. Abdominal incision, fascial suturing, pneumoperitoneum (shoulder pain), and visceral blunt pain (pain at the region of the abdominal surgery) are the common causes of postoperative pain after laparoscopic surgery. NOTES reduces or eliminates abdominal incisions and fascial sutures through the use of natural orifices [4]. The posterior fornix of the vagina is neither somatically innervated nor enveloped in fascia. These anatomic characteristics suggest that it is well suited for an operative approach to the abdominal viscera, which would minimize postoperative pain and expedite recovery. It was shown that transvaginal appendectomy required less postoperative analgesia, produced less pain, reduced the length of the hospital stay, and resulted in a more rapid return to regular activity, as well as a return to work, compared with laparoscopic appendectomy [5, 6]. However, NOTES has some challenges, such as a prolonged operation time, a requirement for additional instruments, and an unusual view of the operation area. Therefore, NOTES procedures, particularly appendectomies, are still limited to highly selected patients with very strict criteria, such as an age range between 18 and 65 [5, 7, 8], an ASA score of I-II [5, 7–10], a BMI < 35 kg/m^2^ [5, 7, 10, 11], short duration of symptoms [9, 10], no history of previous abdominal or pelvic surgery [5], or noncomplicated cases [5, 7, 9, 10]. Here, it is reported that a NOTES appendectomy went beyond those limiting criteria, as evidenced by the patient's age of 66, ASA score of III, BMI of 36 kg/m^2^, and duration of symptoms (48 hours). Despite these potential drawbacks, the transvaginal appendectomy was performed uneventfully, the patient stayed in the hospital fewer than 24 hours, and she did not require any postoperative analgesics.

Standard laparoscopic appendectomies are usually performed using three trocars. A relationship between the patient's BMI and the rate of incisional hernia has been clearly demonstrated. For obese patients, 12 mm trocars lead to a higher risk of hernias (1.9% of patients) that is higher than that for nonobese patients, and the risk of an incisional hernia increases with BMI up to 6% [12]. A single-incision laparoscopic (SILS) appendectomy is another option, although this technique has an increased risk of incisional hernias, even for nonobese patients [13]. Consequently, morbidly obese patients could benefit greatly from the NOTES because it avoids or significantly reduces the number and sizes of abdominal ports that have the potential to produce pain and wound-related complications. In our morbidly obese patient, only a 5 mm umbilical trocar was used without fascia closure, and no wound-related complications were observed.

In morbidly obese patients, the larger labial adipose tissue of the vagina may create some difficulty in accessing the posterior vaginal fornix during the initial insertion of the 12 mm trocar or during the closure of the culdotomy. However, this is a minor drawback, and it can be overcome with the use of larger vaginal retractors. Another limitation is the transvaginal placement of a second trocar. During the procedure, the handles of the trocars cross each other, and this results in an inability to work in the same direction. We overcame this difficulty by inserting a separate grasper alone (without trocar) through the posterior fornix as far as possible from the 12 mm trocar. In this manner, both transvaginal surgical instruments could work against the appendix. Direct insertion of the grasper did not result in any gas leakage or failure of the pneumoperitoneum. The main problem of direct grasper insertion was the inability to switch the grasper with another surgical instrument.

There are very few studies regarding the use of NOTES on morbidly obese patients. Panait et al. [14] reported 17 cases with transvaginal procedures, including 14 cholecystectomies, two ventral hernia repairs, and one appendectomy. However, there were no details regarding the single appendectomy case. The German Registry of NOTES has 551 patients, including 41 morbidly obese patients, although there were no appendectomies [15]. Unfortunately, western populations are becoming heavier, and surgeons are exposed to more obese patients than in the past. Moreover, it is well known that minimally invasive surgeries are more beneficial for obese patients than nonobese patients. Because appendicitis is still the most common cause of emergency abdominal surgery, the use of NOTES for appendicitis in patients with obesity, comorbidity, old age, or complicated appendicitis may be more popular in the future.

In conclusion, to the best of our knowledge, this is the first clinical reported case that focuses on the natural orifice appendectomy in a morbidly obese patient. We can say that morbid obesity does not constitute a great obstacle for transvaginal appendectomy.
